# GABAergic neurons exhibit subtype-specific changes in the developing somatosensory cortex of a rat model of Fragile X Syndrome

**DOI:** 10.3389/fnins.2026.1819200

**Published:** 2026-05-26

**Authors:** Anna Sumera, Abbi Crichton, Peter C. Kind, Christopher Sibley, Sam A. Booker

**Affiliations:** 1 Institute for Neuroscience and Cardiovascular Research, Hugh Robson Building, University of Edinburgh, Edinburgh, United Kingdom; 2 Simons Initiative for the Developing Brain, Hugh Robson Building, University of Edinburgh, Edinburgh, United Kingdom; 3 Patrick Wild Centre, University of Edinburgh, Edinburgh, United Kingdom; 4 Institute of Quantitative Biology, Biochemistry and Biotechnology, School of Biological Sciences, University of Edinburgh, The King’s Buildings, Edinburgh, United Kingdom

**Keywords:** cortical development, Fragile X Syndrome, GABAergic interneuron, parvalbumin interneuron, rat - brain, somatosensory cortex, somatostatin interneuron

## Abstract

**Introduction:**

GABAergic interneurons (IN) are critical for the precise timing and flow of information in cortical circuits. Loss of GABAergic IN function has been suggested as a potential translationally relevant mechanism of neuropathology in Fragile X Syndrome (FXS). Indeed, in rodent models of FXS, some IN populations may display reduced number, while genes associated with other IN type upregulated. However, it remains unknown how these cell populations, and their cell-type specific gene expression patterns are regulated in early development across other mammalian models of FXS.

**Methods:**

Here we utilise an outbred rat model of FXS, in which we have performed single-nucleus RNA sequencing analysis in neonatal development of the somatosensory cortex. We then use immunohistochemistry to measure the number and distribution of neurochemically identified GABAergic IN subtypes from early development until adolescence in the somatosensory cortex.

**Results:**

We find that GABAergic INs in a rat model of FXS display clear evidence of transcriptomic alteration compared to wild-type littermates in early brain development. These effects are most profound in putative parvalbumin INs, but with modest changes in other cell types. From immunohistochemistry, we find that parvalbumin INs appear largely unaffected in density in distribution, but we observe a large upregulation in the number of somatostatin-expressing INs.

**Conclusion:**

GABAergic INs may display cell-type specific transcriptomic regulation in response to the loss of FMRP. Our data suggests minimal alteration of parvalbumin IN density or distribution, but upregulated somatostatin cell numbers. These data partially agree with previous observations in mouse models of FXS.

## Introduction

Fragile X Syndrome (FXS) is a common, single-gene cause of intellectual disability, which often co-occurs with autism, epilepsy, and altered sensory function, such as tactile hypersensitivity (Hagerman et al., 2009). FXS is caused by a CGG repeat expansion within the 5’ UTR of the *FMR1* gene, giving rise to hypermethylation and subsequent transcriptional inactivation of the *FMR1* gene, leading to the loss of its protein product Fragile X Messenger Ribonucleoprotein (FMRP). In mammalian neurons, the RNA binding protein FMRP canonically acts as a brake on translation, loss of which leads to excessive protein synthesis (Osterweil et al., 2010; Bowling et al., 2019). However, beyond this function, FMRP has also been shown to directly interact with ion channels, regulate protein phosphorylation and trafficking to the plasma membrane (reviewed in Deng and Klyachko, 2021). Many of the features of FXS, such as sensory disturbances have been attributed to dysfunction of cortical circuits, including the primary somatosensory cortex (S1; Contractor et al., 2015), which receive rich sensory inputs from the periphery via brainstem and thalamus (Feldmeyer, 2012). As such, defining how loss of FMRP regulates somatosensory cortex development and function is critical to understanding this condition and devising therapeutic strategies.

It is well established, primarily in work from rodents, that appropriate transfer of sensory information in S1 requires excitatory synaptic information to be exquisitely balanced and timed by local GABAergic interneurons (INs, Feldmeyer et al., 2018) Specific to bottom-up sensory inputs, feedforward inhibition is regulated by either fast-spiking parvalbumin (PV) basket and axo-axonic cells, or regular spiking reelin-positive dendritic inhibitory neurons, while feedback and lateral inhibition is regulated by somatostatin (Sst) positive neurons; which together comprise nearly 80% of all GABAergic neurons (Rudy et al., 2011). Impaired function of INs has the potential to dysregulate incoming sensory information and lead to excessive circuit activity, including in rodent models of FXS (Antoine et al., 2019). Indeed, output of PV INs has been shown as elevated in the *Fmr1*^−/y^ mouse model of FXS, both in terms of intrinsic excitability and connectivity (Domanski et al., 2019) and synaptic strength (Antoine et al., 2019). Recently, it has been shown that the density of PV INs may be decreased in *Fmr1*^−/y^ mice, as well as patient samples (Kourdougli et al., 2023). By contrast, the expression of *Sst* mRNA, as well as Sst IN numbers are elevated (Kourdougli et al., 2023) with alterations in Sst IN excitability (Hewitt et al., 2025) and plasticity (Wilson et al., 2026) observed in the hippocampus. However, it remains unknown if these effects are consistent in S1 of a rat model of FXS, and if such changes are present in early brain development.

Loss of FMRP is known to lead to dysregulation of the transcriptome of cortical cells (Donnard et al., 2022; Kourdougli et al., 2023), including many proteins typically associated with GABAergic neuron function and development (Kourdougli et al., 2023). Indeed, assays in excitatory neurons in hippocampus indicate that the differentially actively translated proteins are not exclusively direct targets of FMRP (Thomson et al., 2017). This suggests that constitutive loss of FMRP, as in the case of FXS, leads to functional re-organisation of neurons. This raises the important possibility that some cells are affected more than others, due to unique developmental niches or activity patterns. Determination of cell-type specific modification of neuronal subtypes may reveal targets that could ameliorate FXS pathophysiology in a targeted manner, avoiding off-target effects. To the best of our knowledge, no study has examined GABAergic cell-type specific transcriptome in pre-clinical models of FXS.

In the current study we tested the hypothesis that different GABAergic cell types are selectively vulnerable to the loss of FMRP during early development of the somatosensory cortex of a rat model of FXS, displaying divergent transcriptomic profiles. Using single-nucleus RNA sequencing (snRNA-seq) we show that multiple IN subtypes are present in the developing S1, and that putative PV INs may be the most transcriptomically affected by the loss of FMRP. However, we find a consistent upregulation of *Sst*, supported by a selective increase in the number of Sst-immunoreactive cells in S1, which was maintained into early adolescence. These data reveal distinct cell-type specific modulation of GABAergic cells, which confirms the potential vulnerability of PV INs and compensatory upregulation of Sst INs.

## Materials and methods

### Animals

All experiments were performed using male, outbred Long-Evans Hooded rats at ages P6-38. *Fmr1^+/−^* females were crossed with wild-type males to produce offspring with WT or *Fmr1^−/y^* genotypes in a 1:1 Mendelian ratio in males (Asiminas et al., 2022). Due to the X-linked nature of the model, only male *Fmr1^+/y^* (WT) and *Fmr1^−/y^* littermates were used. Animals were housed on a 12 h light/dark cycle with *ad libitum* access to food and water. All procedures were performed in line with the Animals (Scientific Procedures) Act 1986 under UK Home Office Project License PP2262369.

### snRNA-seq

For 10X snRNA-seq, S1 was acutely dissected from 3 WT and 3 *Fmr1^−/y^* littermate rats at P9, flash frozen on dry ice and stored at −80 °C. S1 nuclei were isolated using a nuclei isolation kit (NUC201, Sigma), according to previous methods (O’Keeffe et al., 2025). The tissue was thawed on ice, then suspended in lysis buffer (PURE lysis buffer, 0.1M DTT, 10% Triton X-100, SUPERaseIn RNase inhibitor (AM2696, Invitrogen) diluted 1:100 in nuclease-free water) and homogenized using insulin needles (324826, BD). 360 μL of sucrose cushion (PURE 2M sucrose solution, sucrose cushion buffer, 0.1M DTT, RNase inhibitor) was added to each sample, and filtered through a sterile 30 μm CellTrics filter (04-004-2326, Sysmex). The filtrate was gently overlaid on top of 200 μL of sucrose cushion. Samples were centrifuged for 45 min at 16,100 g and 4 °C. The supernatant was removed and the remaining pellet was resuspended in 100 μL elution solution (PURE storage buffer, RNase inhibitor) and transferred to a fresh microcentrifuge tube and another 100 μL of the elution solution was added. The samples were then centrifuged for 5 min at 1,000 g, after which the supernatant was discarded and the pellet resuspended in 1% w/v BSA (A9418, Sigma-Aldrich) diluted in Dulbecco’s PBS (14190144, Gibco). FACS sorting and library preparation were performed by the Single-cell and Spatial Biology Facility (Institute for Regeneration and Repair, University of Edinburgh). Nuclei were sorted on BD Fusion Q and BD Aria II flow cytometers, using DAPI for detection. Gating criteria were set based on forward/side scatter to capture singlet nuclei and filter out debris. Quality control was performed on a Luna FX7 automated cell counter (Logos Biosystems). Library preparation was carried out using the Chromium GEM-X scRNA-seq 3′ v4 workflow (10X Genomics), with an input of 30,000 nuclei per sample. Libraries were purified with SPRIselect Beads (Beckman Coulter) and quality controlled using TapeStation and DNA ScreenTape D1000 (Agilent). Sequencing was performed on a NovaSeq 6000 system using a 100 cycle S2 flow cell (Illumina).

### Immunohistochemistry

For immunohistochemistry, rats were sedated with isoflurane then terminally anaesthetised via intraperitoneal injection of sodium pentobarbital (100 mg/kg), then transcardially perfused with phosphate buffered saline (PBS; 0.1M Phosphate Buffer {PB}, 0.9% NaCl) then 4% paraformaldehyde in PB, following which the brains carefully removed, placed in 4% PFA for 3 h and stored in PBS. Perfusion-fixed brains were sectioned coronally into 60 μm sections using a Leica VT1000S vibratome (Leica Biosystems). Prior to labelling the sections were washed in PBS twice for 15 min and placed in blocking solution (10% Normal Goat Serum, 0.3% Triton X-100 and 0.05% NaN_3_ in PBS) for 1 h at room temperature. The sections were incubated with primary antibodies raised against PB (PV-235, mouse monoclonal, SWANT, Bellizona Switzerland), Sst-14 (T-4103, rabbit polyclonal, Peninsula Laboratories, USA), reelin (MAB5364, Millipore, UK), and NeuN (MAB377 and ABN78, mouse monoclonal and rabbit polyclonal; Millipore, UK) for 72 h at 4 °C in PBS containing 5% Normal Goat Serum, 0.3% Triton X-100, 0.05% NaN_3_. The sections were then washed twice in PBS for 15 min and transferred into secondary antibody solution (A11001, A11004, A11008, A11011, goat anti-mouse or anti-rabbit AlexaFluor488 and AlexaFluor588, Invitrogen, USA; diluted in 3% NGS, 0.1% Triton X-100 and 0.05% NaN_3_ in PBS) for 24 h at 4 °C. Following labelling, the sections were washed twice in PBS for 15 min then transferred into 0.1 M PB for at least 1 h and mounted onto glass slides with Vectashield HardSet (H-1400-10, Vector Labs) or Fluoromount G (00-4958-02, Invitrogen) mounting media.

### Confocal imaging and image analysis

In order to estimate the density of IN subpopulations based on the marker labelling (PV, Sst-14, reelin) at P6-38, tiled Z-stack images of the S1 cortical column were taken on a Leica SP8 confocal microscope. Images were taken at 1024 × 1024 resolution with 1 μm step size at 20x magnification. Cortical layers were identified based on the Z-projected NeuN labelling. IN density was measured manually for each cortical layer in FIJI ImageJ using the Cell counter plugin, following the optical disector method (Gundersen et al., 1988; Brown, 2017) and calculated based on the volume of each layer.

### Bioinformatics

Reads from snRNAseq were mapped to a custom-built rat reference genome (Ensembl mRatBN7.2.113) and per cell per gene counts were produced with CellRanger v8.0.1 (10X Genomics). R package SoupX v1.6.2 (Young and Behjati, 2020) was used to estimate and filter out ambient RNA from each sample individually. Count data were merged, normalized, clustered using the Louvain algorithm and quality controlled using the R package Seurat v5.2.1 (Hao et al., 2024). Outliers were identified using the is Outlier function from the scater R package v1.34.0 (McCarthy et al., 2017) and removed based on high mitochondrial gene expression percentage, low/high feature and count numbers as well as high feature/count ratios. Doublets were removed on a per sample basis with scDblFinder R package v1.20.2 (Germain et al., 2022). Transcripts were normalized and re-clustered with PCA using 14 dimensions at 0.04 clustering resolution. Cell type identity was individually assigned using the SCINA R package 1.2.0 (Zhang et al., 2019) based on the expression of established cell type markers (Supplementary Table 1). Inhibitory neuron-specific clusters were isolated, normalized and re-clustered in 9 dimensions at 0.05 clustering resolution. Counts were aggregated on sample level using the aggregateBioVar R package v1.16.0 (Thurman et al., 2021) and pseudobulk differential expression analysis was performed by cell type or subtype with the R package DESeq2 v1.46.0 (Love et al., 2014). *p*-values were adjusted for multiple comparisons using the Benjamini-Hochberg method to control the false discovery rate. Gene ontology (GO) analysis was performed using enrichGO from the R package ClusterProfiler v4.16.6 (Yu et al., 2012) separately for upregulated and downregulated genes using all detected genes as background.

### Experimental design and statistical analysis

For all data, we collected litter-mate paired biological material, whether for snRNA-seq or immunohistochemistry. We based choice of replicate number on previous research (O’Keeffe et al., 2025; Kourdougli et al., 2023), whereby a minimum of 3 biological replicates were collected per group. All sample collection, processing and data analysis of interneuron density images were performed blind to genotype. Unless otherwise indicated, statistical analysis was performed in R using two-way ANOVA with animal as biological replicate. Significance was assumed at *p* < 0.05.

## Results

### snRNA-seq identifies multiple transcriptomic IN subtypes in the developing S1 of WT and *Fmr1^−/y^* rats

**Figure 1:**
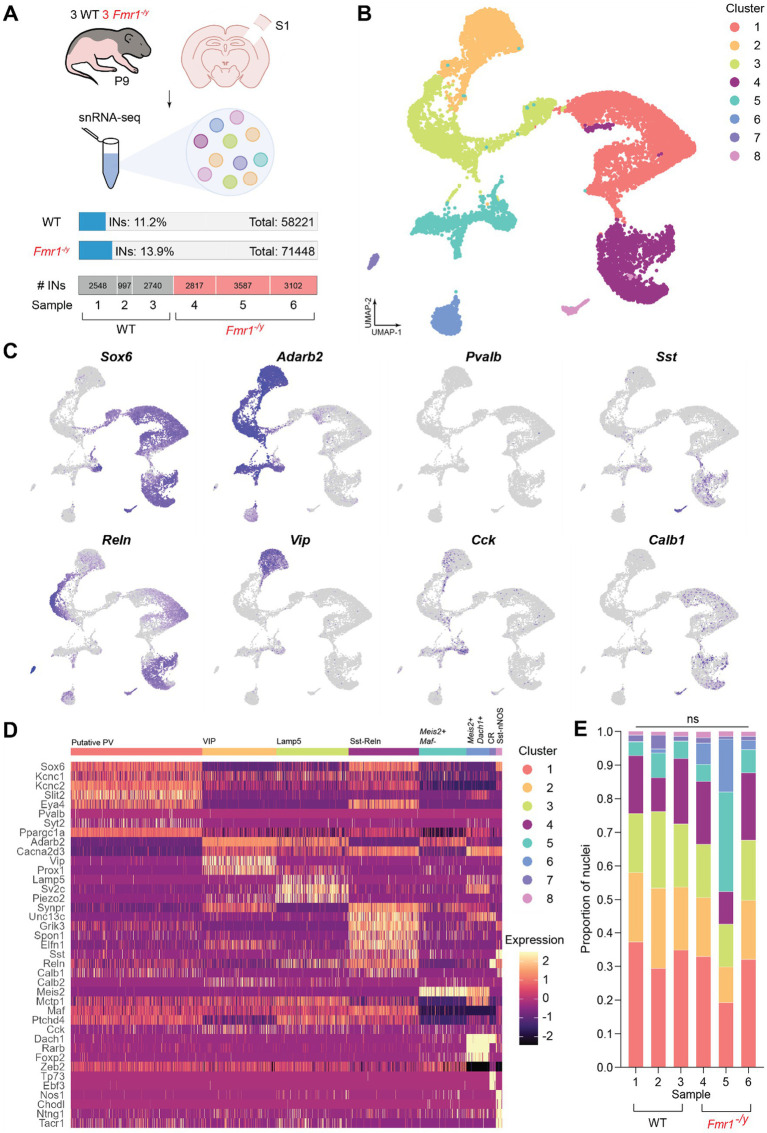
Single nucleus RNA sequencing identifies transcriptomic IN subtypes in the developing S1 of WT and *Fmr1^−/y^* rats. **(A)** Overview of experimental design and sample composition by genotype. **(B)** UMAP projection of IN-specific transcriptomes from WT and *Fmr1^−/y^* S1 at P9. Unbiased clustering identified 8 transcriptomic IN subtype clusters. UMAP—uniform manifold approximation and projection. **(C)** Feature plots of IN gene marker expression for selected genes. Colour intensity indicates normalized expression. **(D)** Heatmap of cluster markers and selected IN gene markers. CR—Cajal–Retzius cells. **(E)** Cluster distribution does not differ by sample (Two-way ANOVA; cluster: *F* = 19.09, *p* < 0.0001; sample: *F* = 9.87 × 10–30, *p* > 0.99).

In order to investigate the IN diversity during early S1 development in a rat model of Fragile X Syndrome, we performed single-nucleus RNA sequencing (snRNA-seq) from isolated S1 cortical sections of 3 WT and 3 *Fmr1^−/y^* littermate rats at postnatal day 9 (Figure 1A), i.e., following the closure of the S1 critical period; concurrent with previously described changes in *Fmr1^−/y^* mice (Gibson et al., 2008, Booker et al., 2019; Domanski et al., 2019). After outlier and doublet filtering, 58,221 cells from WT and 71,448 cells from *Fmr1^−/y^* were obtained, with INs contributing 11.2 and 13.9%, respectively. We detected all major cortical cell types based on their expression of established gene markers and found no differences in the cell type distribution between the genotypes (Supplementary Figure 1). IN-specific transcriptomes were identified based on their expression of *Gad1* and *Gad2*, re-clustered and visualized using UMAP to give rise to 8 distinct clusters (Figure 1B).

Clusters 1, 4 and 8 strongly expressed *Sox6*, suggesting MGE-derived identity (Batista-Brito et al., 2009), while the remaining clusters were predominantly positive for the CGE-derived IN marker *Adarb2* (Figure 1C; Hodge et al., 2019). While *Pvalb* is a marker for the most abundant class of INs, it is typically not expressed in rodent cortex until P14 (de Lecea et al., 1995). Indeed, no *Pvalb* expression was found in any of the IN clusters. Other major IN markers such as *Sst*, *Reln*, *Vip*, *Cck*, and *Calb1* were found to be variably expressed throughout the dataset, suggesting the presence of multiple established classes of INs (Rudy et al., 2011; Tasic et al., 2016; Hodge et al., 2019).

We next examined each cluster’s gene marker expression to identify established IN subtypes (Supplementary Table 2). Cluster 1 was *Sox6^+^* and *Adarb2^−^* indicating MGE-derived identity (Figure 1D), without significant *Sst* expression, thus likely comprising of future PV INs. Additionally, cluster 1 was positive for *Kcnc1* and *Kcnc2*, which have been associated with fast-spiking PV INs (Sekirnjak et al., 1997; Chow et al., 1999), as well as *Ppargc1a*, which is expressed by developing PV INs prior to the onset of *Pvalb* expression and is involved in their maturation (Moissidis et al., 2025). Clusters 2 and 3 expressed *Vip* and *Lamp5, respectively.* Cluster 5 was characterized by *Meis2^+^ Maf^−^* expression while cluster 6 contained *Meis2^+^ Dach1^+^* cells. Cajal–Retzius cells formed cluster 7, with strong *Reln*, *Tp73*, and *Ebf3* expression (Hernández-Acosta et al., 2011; Chiara et al., 2012). Clusters 4 and 8 were both *Sst^+^* and *Reln^+^*, with cluster 4 expressing *Spon1* and *Elfn1*, which have been associated with developing Sst INs (Mayer et al., 2018; Su et al., 2020). Cluster 8 was identified as Sst-nNOS INs based on their specific expression of genes such as *Nos1* and *Chodl1* (Fisher et al., 2024). While sample 5 appeared to have an increased proportion of cells in clusters 5 and 6—likely due to the higher overall number of cells in the sample (Figure 1A)—the proportional representation of different clusters did not significantly differ between samples (Figure 1E) and both genotypes were represented in each cluster (Supplementary Figure 2). Overall, we identified several transcriptomically distinct IN subtypes in the developing S1 of WT and *Fmr1^−/y^* rats.

### Developing INs show broad and subtype-specific changes in gene expression following the loss of FMRP

**Figure 2:**
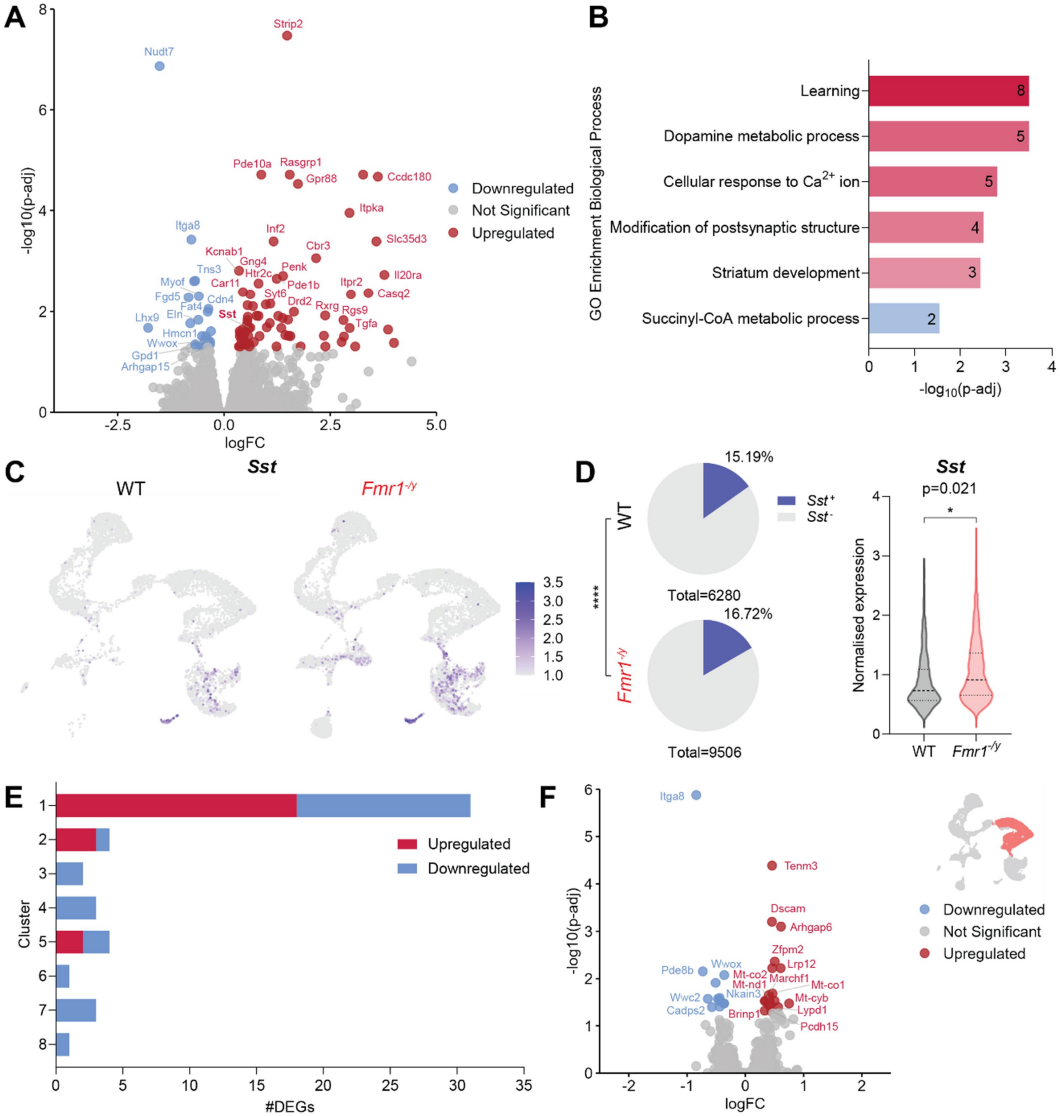
Pseudobulk differential gene expression analysis reveals changes in *Fmr1^−/y^* IN transcriptome. **(A)** Volcano plot of differentially expressed genes (DEGs) in *Fmr1^−/y^* INs. Downregulated genes labelled in blue, upregulated in red. Fmr1 omitted for presentation [−log10(*p*-adj) = 175.5]. **(B)** Gene ontology analysis for genes upregulated (red) and downregulated (blue) *Fmr1^−/y^* INs. Number on the bar and colour intensity indicate the number of genes in each term. **(C)** UMAP feature plot of Sst expression in WT and *Fmr1^−/y^* INs. Colour indicates normalized expression. **(D)** Left—proportion of cells expressing Sst in WT (top) and *Fmr1^−/y^* (bottom) INs. *Fmr1^−/y^* INs have a higher proportion of Sst+ cells compared to WT (Chi-square test; X2 = 17.18, *p* < 0.0001). Right—normalized Sst expression is upregulated in *Fmr1^−/y^* INs. Only cells with normalized Sst expression >0 were included. **(E)** Number of DEGs by IN cluster. **(F)** Volcano plot of DEGs in cluster 1 (putative PV INs). *Fmr1* omitted for presentation [−log(*p*-adj) = 68.2].

We then performed pseudobulk differential gene expression analysis (Lun and Marioni, 2017; Squair et al., 2021) to identify transcriptomic changes in developing INs in the absence of FMRP. *Fmr1^−/y^* INs differentially expressed 93 genes (69 upregulated, 24 downregulated; Supplementary Table 3), with *Strip2*, *Ccdc180*, *Rasgrp1*, *Gpr88*, and *Itpka* among the most upregulated genes (Figure 2A). Gene ontology analysis revealed upregulation in terms related to learning, postsynaptic structure and Ca^2+^ metabolism, as well as downregulation in genes involved in succinyl-CoA metabolism (Figure 2B; Supplementary Table 5), although the low number of genes per GO category warrants cautious interpretation and will require future validation. Interestingly, only 7 out of 93 genes were direct FMRP targets (Darnell et al., 2011; Supplementary Table 3), suggesting transcriptomic-level compensation following loss of FMRP. Notably, *Sst* was found to be upregulated in the broad IN population in *Fmr1^−/y^* rats (Figure 2C), consistent with previous reports in mice (Kourdougli et al., 2023). This was underlain by a minor increase in the proportion of *Sst^+^* cells in *Fmr1^−/y^*, as well as higher levels of average *Sst* expression relative to WT (Figure 2D).

We next asked whether the transcriptomes of developing *Fmr1^−/y^* rat INs could be differentially affected by subtype. Indeed, we found that cluster 1, i.e., putative PV INs, differentially expressed the highest number of genes (31; Supplementary Table 4) compared to fewer than 5 in the remaining subtypes (Figure 2E), suggesting that immature PV INs may be uniquely affected by the loss of FMRP on a transcriptomic level. These changes included upregulation in genes involved in establishing and organizing neuronal projections and connectivity (*Tenm3, Dscam, Lrp12, Pcdh15, Brinp1*; Figure 2F), as well as mitochondrial genes (*Mt-co2, Mt-co1, Mt-nd1, Mt-cyb*). In conclusion, *Fmr1^−/y^* inhibitory neurons show transcriptomic changes specific to the putative PV IN subtype implying altered synaptic function and connectivity.

### Sst INs display increased density across supragranular S1 layers in early development, while PV and Reln INs are unaffected

**Figure 3:**
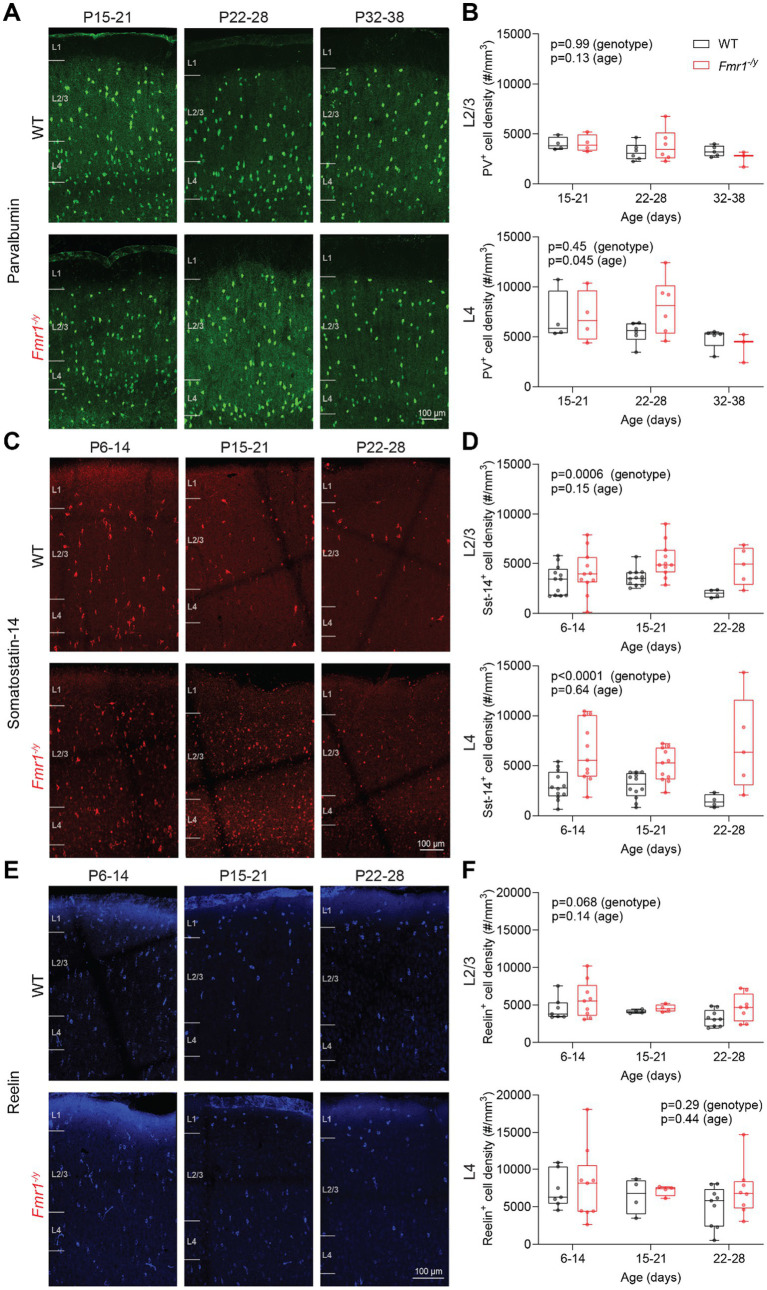
Sst-14, but not PV and reelin-expressing INs have increased density in *Fmr1^−/y^* rat S1 during development. **(A)** Representative PV+ cell density images from WT and *Fmr1^−/y^* S1 at P15-38 **(B)**. **(C)** *Fmr1^−/y^* rats show no change in L2/3 PV+ cell density (Two-way ANOVA; age: *F* = 2.25, *p* = 0.13; genotype: *F* = 1.84 × 10^–5^, *p* = 0.99; age × genotype: *F* = 1.05, *p* = 0.37). In L4 the PV+ cell density decreases with age, but does not differ between WT and *Fmr1^−/y^* (Two-way ANOVA; age: *F* = 3.58, *p* = 0.045; genotype: *F* = 0.58, *p* = 0.45; age × genotype: *F* = 1.81, *p* = 0.19). WT: *n* = 15 animals; *Fmr1^−/y^*: *n* = 13 animals. C. Representative Sst-14+ cell density images from WT and *Fmr1^−/y^* S1 at P6-28. **(D)** Sst-14+ cell density is increased in *Fmr1^−/y^* rats in L2/3 (Two-way ANOVA; age: *F* = 1.96, *p* = 0.15; genotype: *F* = 13.28, *p* = 0.0006; age × genotype: *F* = 1.34, *p* = 0.27) and L4 (Two-way ANOVA; age: *F* = 0.45, *p* = 0.64; genotype: *F* = 29.97, *p* < 0.0001; age × genotype: *F* = 2.07, *p* = 0.14). WT: *n* = 28 animals; *Fmr1^−/y^*: *n* = 27 animals. **(E)** Representative reelin+ cell density images from WT and *Fmr1^−/y^* S1 at P6-28. **(F)**. *Fmr1^−/y^* rats show a trend toward an increase in reelin+ cell density in L2/3 (Two-way ANOVA; age: *F* = 2.078, *p* = 0.14; genotype: *F* = 3.55, *p* = 0.068; age × genotype: *F* = 0.25, *p* = 0.78), but no difference in L4 (Two-way ANOVA; age: *F* = 0.84, *p* = 0.44; genotype: *F* = 1.16, *p* = 0.29; age × genotype: *F* = 0.25, *p* = 0.78). WT: *n* = 20 animals; *Fmr1^−/y^*: *n* = 21 animals.

In order to determine whether the development of selected IN populations is affected by the loss of FMRP, we investigated the developmental distribution of major IN subtypes in the supragranular layers of S1 by immunolabelling for PV, Sst and reelin, which altogether account for ~80% of all cortical INs (Rudy et al., 2011). Since PV is not expressed in the rodent cortex until P14 (de Lecea et al., 1995), we examined PV cell density at P15-38, while Sst and Reelin cell densities were measured at P6-28, as their expression is known to be present from earlier developmental stages (Alcántara et al., 1998; Lee et al., 1998). Surprisingly, we found no changes in the density of PV-expressing cells in L2/3 and L4 (Figures 3A,B) between P15 and P38, suggesting that their distribution is unaffected in the first postnatal month despite the transcriptomic-level changes in this subtype, contrary to previous findings in *Fmr1^−/y^* mice and FXS individuals (Selby et al., 2007; Lee et al., 2019; Kourdougli et al., 2023). In L4, the PV^+^ cell density decreased with age in both genotypes, potentially reflective of the elimination of excess INs that occurs during early postnatal development (Southwell et al., 2012; Priya et al., 2018). In contrast, we found that Sst^+^ cell density was significantly increased in the *Fmr1^−/y^* rats compared to WT in L2/3 and L4 regardless of age (Figures 3C,D), in line with the transcriptomic upregulation of *Sst* in *Fmr1^−/y^* INs during development. Reelin^+^ cells showed a tendency toward increased density in L2/3 but not L4 of *Fmr1^−/y^*, and no effect of age (Figures 3E,F). Overall, we found that in the first month of postnatal development the S1 of *Fmr1^−/y^* rats shows changes to IN diversity that are specific to the Sst^+^ subtype.

## Discussion

In the current study, we provide evidence that GABAergic cells identified in early brain development using transcriptomic and immunolabelling approaches display cell type-specific alterations in the *Fmr1*^−/y^ rat model of FXS. Specifically, we show that putative PV INs display the greatest transcriptomic divergence from WT littermates, but are largely preserved in number and localisation in early development. By contrast, Sst INs appear to possess minimal transcriptomic differences, but display excessive numbers throughout the upper layers of S1. These data indicate that cellular and circuit deficits attributed to INs in FXS may originate early in development in a cell-type specific manner, and not hard-wired from conception– rather developing in response to ongoing circuit activity.

### Altered GABAergic transcriptomes in the developing S1 of the *Fmr1^−/y^* rat

The postnatal brain experiences broad changes in GABAergic circuitry over the first postnatal weeks of life in rodents. Following migration of GABAergic cells from the ganglionic eminences to the cortex, neurons undergo activity dependent apoptosis, formation of local and long-range synaptic connections, and maturation of physiological properties (Southwell et al., 2012; Butt et al., 2017; Priya et al., 2018). As such, defining cell type-specific differences in diversity in early life of neurodevelopmental conditions (such as Fragile X Syndrome) may reveal novel targets for therapeutic intervention.

In mouse models of FXS, PV INs have been shown to display alterations in cellular function, synaptic connectivity (Gibson et al., 2008; Domanski et al., 2019), with their function regulated to maintain circuit-wide homeostasis (Antoine et al., 2019). Consistent with this, recent research has suggested a considerable (>70%) loss of PV INs in postnatal S1 from the *Fmr1^−/y^* mouse, using a fluorescent reporter line driven by transcriptional regulators (Kourdougli et al., 2023). In contrast, we observe no apparent difference in the number of PV INs in *Fmr1^−/y^* rat S1 up until 38 days postnatally. One explanation for this discrepancy is that *Pvalb* mRNA and protein is minimally expressed until 2–3 weeks postnatally in the rat (Alcántara et al., 1993; de Lecea et al., 1995) and PV immunoreactivity peaks potentially even later in mice (del Rio et al., 1994). Considering the developing S1 undergoes delayed maturation in *Fmr1^−/y^* mice (Harlow et al., 2010), a reduced PV IN density could also be reflective of a delay in PV expression rather than a meaningful deficit in IN diversity. However, the loss of PV INs has been reported previously, with earlier studies in adults also noting reductions in PV INs and PV labelling intensity in 1-year old mice (Selby et al., 2007) and 57+ year-old humans (Kourdougli et al., 2023; Juarez et al., 2024). Whether this IN population is vulnerable in later life of FXS individuals is an important question that remains unexplored, although FMRP may regulate brain function in aging (Ribeiro et al., 2026). How PV INs with altered transcriptomic signatures integrate into developing brain circuits in individuals with FXS is an important question that likely has direct implications for early life treatment, especially given these INs undergo dramatic alterations in function and connectivity over early brain developmental (Doischer et al., 2008; Micheva et al., 2021). Most interestingly, even at this early developmental stage few of the transcriptionally dysregulated genes appear to be previously identified FMRP targets (Brown et al., 2001; Darnell et al., 2011). This broadly agrees with other studies in *Fmr1^−/y^* mice, where few translated genes are FMRP targets in hippocampal pyramidal cells (Thomson et al., 2017). Therapeutically, this is a fundamentally important point, as even at the earliest developmental stages neuronal dysfunction may have extended beyond just direct effects of FMRP. Thus treatments based on postnatal research may only ever target emergent phenotypes and not the causal nature of FXS.

Nevertheless, PV IN function has been consistently observed as impaired in *Fmr1^−/y^* mice, displaying cellular hypoexcitability in acute brain slices (Domanski et al., 2019) and *in vivo,* with targeted activation of PV INs ameliorating behavioural dysfunction (Kourdougli et al., 2023). Furthermore, selective loss of FMRP in PV INs may lead to elevated local protein synthesis and anxiety-like behaviour (Kalinowska et al., 2022)—both of which are observed in constitutive knock-out mice. Such evidence for impaired PV IN function is consistent with the increased DEG expression we observed in putative PV INs. Specifically, we observe upregulation of genes that may have direct therapeutic relevance. For instance, *Dscam*—a cell adhesion molecule involved in axon guidance, dendritic branching, and synaptic formation (reviewed in Hizawa et al., 2024) is significantly upregulated in putative PV INs. Increased *Dscam* is associated with intellectual disability and inhibitory synapse development, which may contribute to impaired PV function. Similarly, *Pde8b,* encoding a phosphodiesterase which regulates intracellular cyclic-AMP levels, is downregulated, which may contribute to impaired excitability of PV INs. One key finding from our data is that loss of FMRP appears to preferentially affect PV INs in early brain development. This is in good agreement with earlier work where FMRP was selectively removed from PV and Sst INs, the latter having minimal effect on behaviour or protein synthesis (Kalinowska et al., 2022). Together with our data, this confirm that FMRP expression in PV INs is crucial to neonatal S1 circuit function.

### Upregulation of SST cell numbers in developing S1 of the *Fmr1^−/y^* rat

One of the most prominent features we observed in our GABAergic snRNA-seq data, was an upregulation of the neuropeptide *Sst*, which was confirmed by an increased density of Sst-14 immunolabelling across L2/3 and L4 of S1—with limited changes in PV and reelin populations. This is not entirely unexpected, as previous bulk RNA-seq also observed increased *Sst* expression, as well as increased Sst immunolabelled somata in *Fmr1^−/y^* mice (Kourdougli et al., 2023). Contrastingly, immunolabelling using different antibodies failed to detect an obvious difference in *Fmr1^−/y^* mice (Paluszkiewicz et al., 2011). Interestingly, we found very few DEGs in Sst INs in snRNA-seq data, suggesting that this population of cells is either largely functionally unaffected by loss of FMRP, or any changes occur later, such as upregulated LTP (Wilson et al., 2026) and reduced excitability (Hewitt et al., 2025) reported from hippocampal Sst INs in adult *Fmr1^−/y^* mice.

In line with the upregulation of *Sst*, we found an increase in the number of Sst-14 immunoreactive cells in S1 of the *Fmr1^−/y^* rats over development. The proportional increase is a greater than expected from gene transcript levels and estimated numbers of transcriptomically identified Sst INs. One potential explanation for this may be transient upregulation of *Sst* mRNA following seizure like activity (Hashimoto and Obata, 1991), which leads to transient expression of Sst-14 in pyramidal cells of the subiculum. Given the excitatory neurons of S1 in *Fmr1*^−/y^ mice are known to be hyperexcitable early in development (Domanski et al., 2019; Booker et al., 2019) with enhanced UP-state duration (Gibson et al., 2008), it is tempting to speculate that elevated Sst-14 immunoreactivity may be a direct consequence of such hyperexcitability. Furthermore, other IN subtypes, including PV INs, are known to express Sst-14 under periods of neuronal stress (Bering et al., 1995). Whether the elevated Sst-14 immunolabelling we observe reflects a greater neuromodulatory potential of S1 in *Fmr1*^−/y^ rats, homeostatically upregulated to prevent the emergence of pathological activity remains unexplored. The functional consequences of elevated Sst expression in humans with FXS have yet to be explored, but our data add to a growing body of evidence for alterations to neurochemical expression that may lead to circuit level dysfunction (Real et al., 2011; Kourdougli et al., 2023)—which may offer interesting avenues to understand neurodevelopmental conditions more generally.

### Technical limitations

A key limitation of the current study is that we only sampled one age point of development in our transcriptomic analysis. However, the purpose of this study was to examine whether INs display apparent alterations in transcriptome prior to significant maturation of cortical circuits – such as development and maturation of GABAergic synapses (Huang, 2009), maturation of neuronal excitability (McCormick and Prince, 1987), or activity dependent cell-death (Southwell et al., 2012; Priya et al., 2018); potentially reflecting some of the earliest changes in function in FXS. Indeed, by measuring these features at P9, we are sampling an age that may loosely approximate with the perinatal period in humans (Sengupta, 2013), thus reflecting the first possible occasion for postnatal diagnosis and therapeutic intervention. Nevertheless, it is highly likely that as GABAergic cells mature, they display increased transcriptomic divergence from WT in *Fmr1*^−/y^ models, as reflected by recent bulk RNA-sequencing efforts from the murine S1 (Kourdougli et al., 2023).

Another limitation relates to the chosen method of transcriptomic measurement—snRNA-seq. Indeed, the sampling of single cells or nuclei leads to a low (20–50%) coverage of transcripts in individual neurons (Bakken et al., 2018). Thus, it is likely that INs that are highly represented in S1 (e.g., PV INs ~ 50% of INs—Rudy et al., 2011) may favour greater detection of divergent transcripts—simply due to greater statistical power. Thus, in future studies it would be important to both validate the relative expression of DEGs in individual IN subtypes, including those with borderline statistical significance using targeted approaches (e.g., quantitative RT-PCR). However, such approaches would also require isolation of given GABAergic cell-types prior to quantification—which may be difficult given the early developmental stage of different INs—e.g., PV INs.

Finally, cortical GABAergic INs are highly diverse anatomically, physiologically, and transcriptomically (Gouwens et al., 2019)—but with many cell-types displaying a high degree of evolutionary conservation (Chartrand et al., 2023). Nevertheless, how well do commonly chosen markers for GABAergic cells in mature neurons (Rudy et al., 2011) map onto developing brains? We have approximated PV IN identity in the current study based on multiple convergent genes, but substantive evidence for early developmental identity of diverse cell populations is scant—beyond whether they derive from medial or caudal ganglionic eminences (Bandler et al., 2017). Likewise, reelin is a well described marker for neurogliaform type neurons in mature networks, but in early development may also include progenitor cells (e.g., Cajal Retzius cells), while many INs, including Sst INs, undergo activity-dependent apoptosis (Southwell et al., 2012; Priya et al., 2018). We have made a best estimate judgement of cell type, however future identification may better define cell types and thus require re-examination of the data we have generated (available in Supplementary material and online).

## Conclusions and outlook

In the current study we provide evidence that PV INs may be transcriptomically vulnerable in FXS, but may not be altered in number as previously suggested in mouse models. However, we confirm that Sst INs are upregulated in the S1, as suggested from earlier studies, but that these cells may have largely unaffected transcriptomes. Further studies need to consider the functional aspects of GABAergic INs beyond PV INs when considering circuit dysfunction in FXS and other neurodevelopmental conditions.

## Data Availability

snRNA-seq dataset is available on GEO under the accession code GSE331164.
