# Supplementary material for: GABAergic neurons exhibit subtype-specific changes in the developing somatosensory cortex of a rat model of Fragile X Syndrome
Source: Front Neurosci. 2026 May 26;20:1819200. doi: 10.3389/fnins.2026.1819200 (PMC13246699; doi:10.3389/fnins.2026.1819200)
Supplement: Supplementary file 1 [file Data_Sheet_1.PDF]

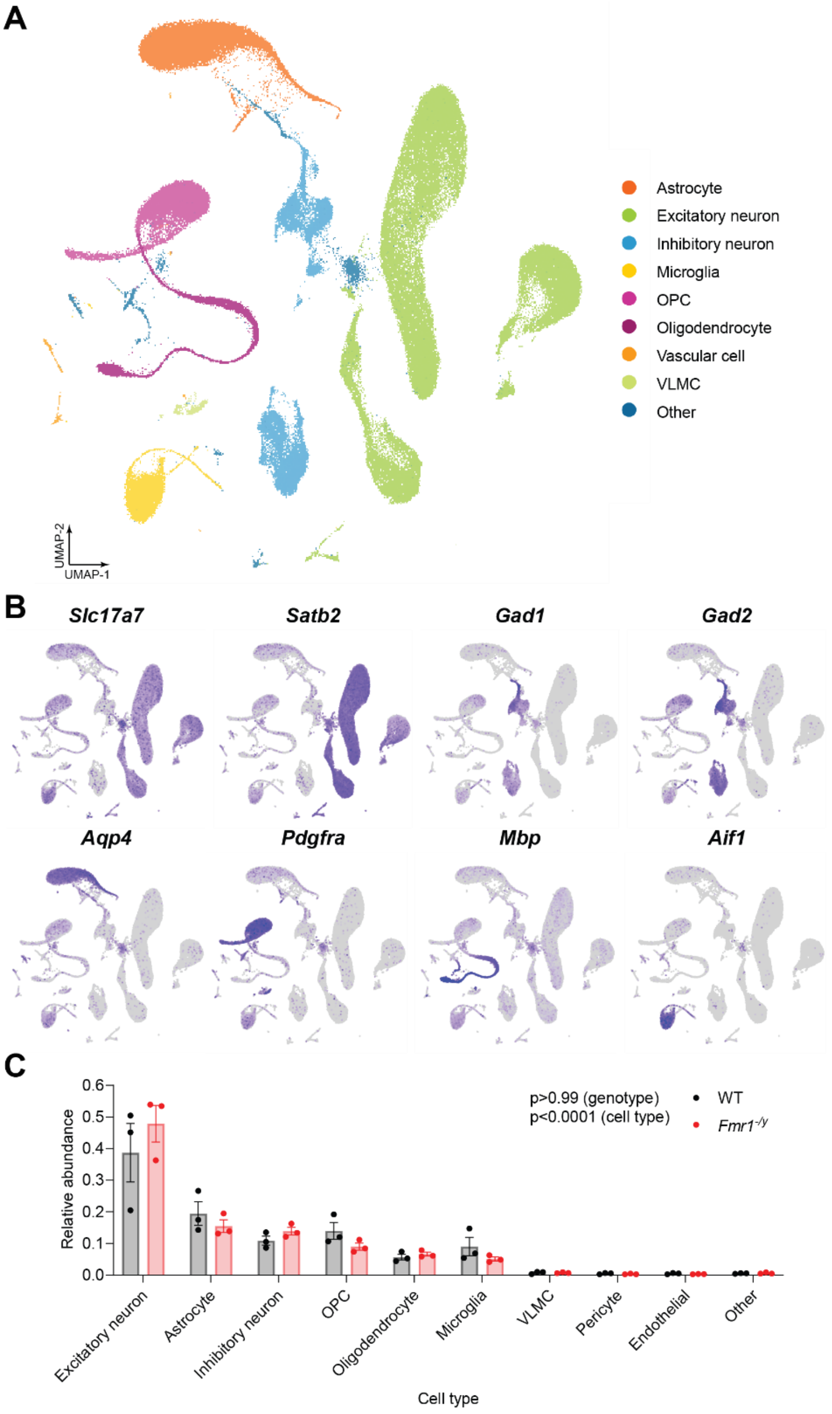

Supplementary Figure 1. Fmr1$^{-/y}$ rats show no changes in cortical cell type distribution. A. UMAP projection of S1 nuclei from WT and Fmr1$^{-/y}$ rat S1 at P9. OPC – oligodendrocyte progenitor cell; vascular cell – pericytes and endothelial cells; VLMC – vascular leptomeningeal cell B. Feature plots of selected cell type marker genes. Colour intensity indicates normalized expression. C. Relative proportions of cells are not different between WT and Fmr1$^{-/y}$ rats regardless of cell type (Two-way ANOVA; cell type: F=43.53, p<0.0001; genotype: F=0.00, p>0.99; interaction: F=1.05, p=0.42).

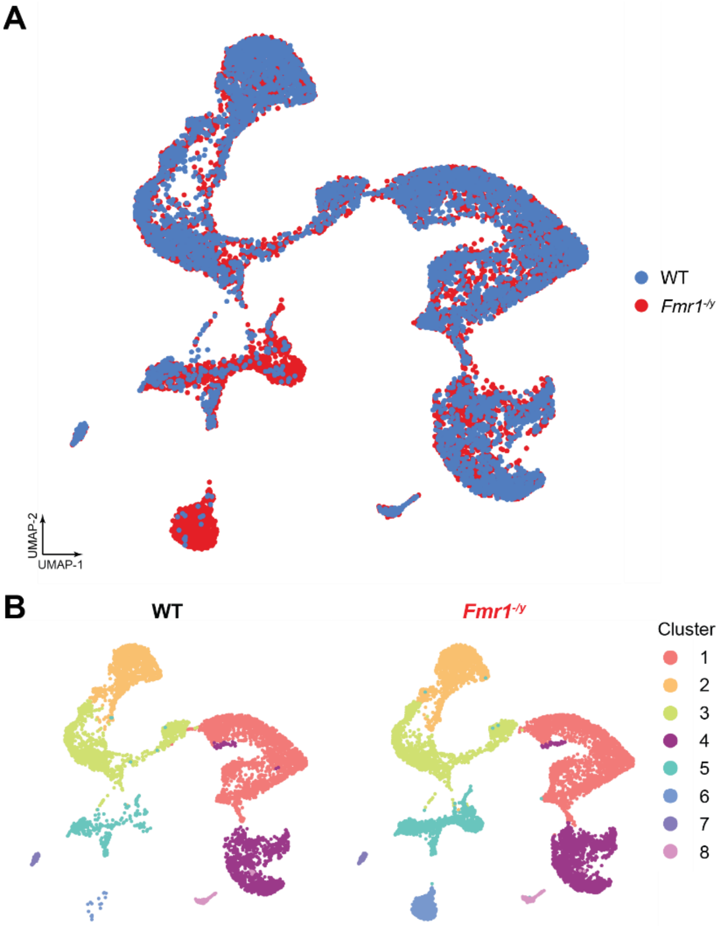

Supplementary Figure 2. Both genotypes are represented across the UMAP projection with no genotype-specific clusters present. A. UMAP projection of interneuron transcriptomes from WT (blue) and $Fmr1^{-/y}$ rats (red). B. UMAP projection of WT (left) and $Fmr1^{-/y}$ (right) interneuron transcriptomes by cluster.

Supplementary Table 1. List of marker genes used to identify cell types in SCINA.

<table>
<tr><th colspan="2">Cell type</th><th>Markers</th></tr>
<tr><td colspan="2">Excitatory neuron</td><td>Slc17a7 Satb2</td></tr>
<tr><td colspan="2">Inhibitory neuron</td><td>Gad1 Gad2</td></tr>
<tr><td colspan="2">Astrocyte</td><td>Aqp4 Gfap Aldh1l1</td></tr>
<tr><td colspan="2">OPC</td><td>Pdgfra Sox10</td></tr>
<tr><td colspan="2">Oligodendrocyte</td><td>Mog Mbp Plp1</td></tr>
<tr><td colspan="2">Microglia</td><td>Csf1r P2ry12 Aif1</td></tr>
<tr><td rowspan="2">Vascular cell</td><td>Endothelial cell</td><td>Vwf Flt1</td></tr>
<tr><td>Pericyte</td><td>Pla1a Cox4i2</td></tr>
<tr><td colspan="2">VLMC</td><td>Dcn Lum Ptgds</td></tr>
</table>

Supplementary Table 2. List of cluster markers used to identify interneuron subtypes.

Cluster	Identity	Cluster marker genes
1	Putative PV	*Sox6*$^{+}$ *Slit2*$^{+}$ *Eya4*$^{+}$ *Kcnc1*$^{+}$ *Kcnc2*$^{+}$ *Adarb2*$^{-}$ *Cacna2d3*$^{-}$
2	VIP	*Vip*$^{+}$ *Adarb2*$^{+}$ *Prox1*$^{+}$
3	Lamp5	*Lamp5*$^{+}$ *Sv2c*$^{+}$ *Piezo2*$^{+}$ *Synpr*$^{-}$
4	Sst-Reln	*Sst*$^{+}$ *Unc13c*$^{+}$ *Grik3*$^{+}$ *Spon1*$^{+}$ *Elfn1*$^{+}$
5	*Meis2*$^{+}$ *Maf*$^{-}$	*Meis2*$^{+}$ *Mctp1*$^{-}$ *Maf*$^{-}$ *Ptchd4*$^{-}$
6	*Meis2*$^{+}$ *Dach1*$^{+}$	*Meis2*$^{+}$ *Dach1*$^{+}$ *Rarb*$^{+}$ *Foxp2*$^{+}$ *Zeb2*$^{-}$
7	Cajal-Retzius	*Tp73*$^{+}$ *Ebf3*$^{+}$
8	Sst-nNOS	*Sst*$^{+}$ *Nos1*$^{+}$ *Chodl*$^{+}$ *Ntng1*$^{+}$ *Tacr*$^{+}$

Supplementary Table 3. List of DEGs in Fmr1$^{-/y}$ *interneurons. FMRP targets are highlighted in blue.*

Gene	log2FoldChange	lfcSE	padj
Fmr1	-7.21685	0.251979	3.20E-176
Strip2	1.483222	0.214204	3.34E-08
Nudt7	-1.51518	0.22734	1.35E-07
MGC94891	3.270579	0.561631	1.93E-05
Pde10a	0.876051	0.15163	1.93E-05
Rasgrp1	1.543659	0.265826	1.93E-05
Ccdc180	3.619461	0.631097	2.12E-05
Gpr88	1.738096	0.307305	2.96E-05
Itpka	2.951369	0.546352	0.000112
Itga8	-0.76956	0.148964	0.000365
Slc35d3	3.58303	0.699562	0.000396
Inf2	1.164034	0.227505	0.000396
Cbr3	2.165864	0.437152	0.000851
Kcnab1	0.352811	0.073093	0.001512
Il20ra	3.771412	0.789975	0.001837
Penk	1.388121	0.292144	0.001926
Pde1b	1.239478	0.262962	0.002186
Tns3	-0.67884	0.144948	0.002393
LOC134478878	-0.70506	0.151086	0.00246
Htr2c	0.810748	0.174911	0.002721
Car11	0.444749	0.097918	0.004048
Casq2	3.396022	0.751057	0.004256
Ddc	2.984881	0.664633	0.004507
Gng4	0.617383	0.137387	0.004507
Myof	-0.59244	0.132708	0.004905
Fgd5	-0.83405	0.187673	0.005179
Echdc2	1.089016	0.249019	0.00692
Syt6	0.982452	0.225504	0.007197
Chn2	0.55384	0.127571	0.00745
Phyhip	0.679935	0.157248	0.007797
Cdh4	-0.3661	0.085358	0.008838

Fat4	-0.38293	0.090094	0.010049
Drd2	1.643233	0.386976	0.010049
Rxrg	2.379063	0.566312	0.01193
Gprin3	0.765227	0.182553	0.012068
Frem2	0.810012	0.193835	0.012212
Itpr2	1.275744	0.305459	0.012212
Slc4a4	0.560356	0.134657	0.012706
Erf	1.379892	0.333082	0.013426
Spata13	1.503795	0.364975	0.014439
Eln	-0.6053	0.14719	0.014578
Rgs9	2.808967	0.685286	0.014752
Fras1	0.551801	0.134631	0.014752
ENSRNOG00000062316	1.078255	0.26554	0.016977
Hmcn1	-0.79412	0.195885	0.017075
Schip1	0.54219	0.134109	0.017515
Icam5	1.004038	0.251011	0.02057
Rasgef1b	0.616121	0.154377	0.020918
Lhx9	-1.78665	0.448742	0.021107
Sst	0.579172	0.14555	0.021107
Tgfa	2.955078	0.74399	0.021197
B3gnt2	1.345012	0.338893	0.021197
Mt-cyb	0.429827	0.108895	0.022772
Neurod2	3.857911	0.978515	0.022779
Spetex2gl1	-0.30711	0.078327	0.024479
Cplx2	0.367326	0.09383	0.024659
Marcks	0.494988	0.12692	0.025755
Mt-nd1	0.387501	0.099784	0.027104
Cchcr1	1.442613	0.373741	0.02934
Strn	0.567249	0.147573	0.030166
Fos	1.51911	0.395022	0.030166
Sh3rf2	2.349565	0.611711	0.030166
Septin9	-0.51818	0.135194	0.03068
Rps16	0.45154	0.118194	0.030693
Emid1	0.83528	0.219006	0.030693
Drd1	1.561528	0.409152	0.030693
Cacna2d3	0.37217	0.097231	0.030693
Wwox	-0.42863	0.112154	0.030693
Asb2	2.820781	0.741319	0.031354
Jph1	-0.38198	0.101629	0.036229
Slc35f3	0.466036	0.123855	0.036229
Mt-co2	0.405292	0.107752	0.036229
Nxph2	-0.46149	0.123283	0.037963
Necab1.1	-0.32442	0.087131	0.040011
Chst11	0.701636	0.188613	0.040011
Kremen1	2.765798	0.743287	0.040011
Rarb	3.996319	1.078467	0.041808

Bcl7c	-0.32794	0.088961	0.044388
LOC102551114	0.585433	0.15892	0.044388
Gpd1	-0.67701	0.184214	0.044795
Rpl18a	0.516393	0.14043	0.044795
Dnah11	-0.6639	0.181839	0.048615
Slc22a3	3.084939	0.847368	0.049358
Suclg2	-0.43739	0.120611	0.049358
ENSRNOG00000067970	0.418089	0.115032	0.049358
Arhgap15	-0.45586	0.125863	0.049358
Grpr	-0.65047	0.179954	0.049358
Six3	2.384623	0.658052	0.049358
Tuba1a	0.350067	0.096663	0.049358
Ccdc3	1.804039	0.498946	0.049358
Gpr6	1.234094	0.340942	0.049358
Cpne5	0.593477	0.163817	0.049358
Mt-co1	0.374542	0.1033	0.049358

Supplementary Table 4. List of DEGs in cluster 1 (putative PV) interneurons.

**Gene**	**log2FoldChange**	**lfcSE**	**padj**
Fmr1	-7.28743	0.40363	5.74E-69
Itga8	-0.8421	0.134055	1.33E-06
Tenm3	0.458567	0.081079	4.10E-05
Dscam	0.456653	0.089326	0.000631
Arhgap6	0.61247	0.121881	0.000798
Zfpm2	0.504955	0.10856	0.004356
Lrp12	0.606474	0.134041	0.005997
Mt-co2	0.461808	0.102046	0.005997
Pde8b	-0.72969	0.163409	0.007039
Wwox	-0.36253	0.082306	0.008396
AABR07027925.1	-0.51044	0.11869	0.012273
Mt-co1	0.466541	0.112076	0.020775
Marchf1	0.403343	0.097674	0.022171
Nkain3	-0.44018	0.107939	0.025719
Wwc2	-0.47034	0.116917	0.026809
LOC134478878	-0.64293	0.15932	0.026809
Mt-nd1	0.412593	0.102196	0.026809
Ralyl	0.341162	0.085874	0.029828
Lancl3	-0.44498	0.112142	0.029828
Tmem132d	0.333721	0.084371	0.029828
Mt-cyb	0.504341	0.127767	0.029828
Adgrv1	-0.36314	0.093443	0.033628
Necab1.1	-0.36849	0.094818	0.033628
Lypd1	0.751991	0.192932	0.033628
Chn2	0.433273	0.112002	0.03473
Pcdh15	0.399847	0.103809	0.035752

Plxdc2	-0.43969	0.115126	0.039314
Grm1	0.562565	0.14791	0.040204
Cadps2	-0.57392	0.151192	0.040204
Brinp1	0.332842	0.088876	0.047662
Myo10	0.445606	0.119513	0.049255

Supplementary Table 5. GO analysis results

**Description**	**GO ID**	**p-adjust**	**Fold enrichment**	**# Genes**	**Gene names**
Learning	GO:0007612	0.000308	11.84113	8	*Gpr88 Pde1b Htr2c Drd2 Neurod2 Fos Drd1 Tuba1a*
Dopamine metabolic process	GO:0042417	0.000308	30.99047	5	*Pde1b Htr2c Ddc Drd2 Drd1*
Cellular response to Ca^{2+} ion	GO:0071277	0.001533	17.297	5	*Itpka Neurod2 Fos Tuba1a Cpne5*
Modification of postsynaptic structure	GO:0099010	0.003049	23.334	4	*Itpka Marcks Strn Drd1*
Striatum development	GO:0021756	0.00358	38.80545	3	*Drd2 Drd1 Rarb*
Succinyl-CoA metabolic process	GO:0006104	0.028377	195.0333	2	*Nudt7 Suclg2*
